# The Influences of Visceral Fat Area on the Sites of Esophageal Mucosal Breaks in Subjects with Gastroesophageal Reflux Diseases

**DOI:** 10.1155/2019/9672861

**Published:** 2019-02-17

**Authors:** Ji Hyung Nam, Eirie Cho, Jeung Sook Kim, Eun-Cheol Park, Jae Hak Kim

**Affiliations:** ^1^Department of Internal Medicine, Dongguk University College of Medicine, Ilsan Hospital, Goyang, Republic of Korea; ^2^Department of Medicine, Graduate School, Yonsei University, Seoul, Republic of Korea; ^3^Department of Radiology, Dongguk University College of Medicine, Ilsan Hospital, Goyang, Republic of Korea; ^4^Department of Preventive Medicine and Institute of Health Services Research, Yonsei University College of Medicine, Seoul, Republic of Korea

## Abstract

**Background:**

Central obesity is suggested as a risk factor for gastroesophageal reflux diseases. The aim of this study was to evaluate the influences of a visceral fat area on the site of mucosal breaks in the esophagogastric junction (EGJ).

**Methods:**

Subjects who underwent abdomen-computerized tomography and esophagogastroduodenoscopy for screening on the same day were evaluated between 2007 and 2016. We enrolled 178 subjects who had erosive esophagitis (LA classifications A-D). Abdominal obesity was evaluated by measuring visceral adipose tissue (VAT), subcutaneous adipose tissue (SAT), VAT-to-SAT ratio, total adipose tissue (TAT), body mass index (BMI), and waist circumference (WC).

**Results:**

The lesser curvature (LC) of EGJ was the most frequent site of mucosal breaks (104 cases, 58.4%). BMI, WC, VAT, the VAT-to-SAT ratio, and TAT were higher in the LC group. In multivariate analysis, higher VAT (odds ratio (OR) 2.90, 95% confidence interval (CI) 1.18 to 7.13, 3rd vs. 1st quartile, *P* = 0.021; OR 3.63, 95% CI 1.44 to 9.10, 4th vs. 1st quartile, *P* = 0.006) and the VAT/SAT ratio (OR 2.91, 95% CI 1.11 to 7.61, 3rd vs. 1st quartile, *P* = 0.03; OR 3.02, 95% CI 1.17 to 7.83, 4th vs. 1st quartile, *P* = 0.023) were significantly associated with mucosal breaks in the LC group. However, BMI, WC, and TAT were not significant in the multivariate analysis.

**Conclusion:**

The VAT and the VAT/SAT ratio were significantly associated with the mucosal breaks in the LC of EGJ. Visceral obesity could influence the location of the mucosal breaks on EGJ.

## 1. Introduction

The symptom-based gastroesophageal reflux disease (GERD) and endoscopic reflux esophagitis have increased in Asian countries [1]. GERD is related to several clinical conditions such as smoking, hiatal hernia, and obesity [2, 3]. Metabolic syndrome also increases the risk of GERD [4, 5]. It has shown an upward trend in obesity and metabolic syndrome, which reflects the recent socioeconomic development and Westernized lifestyle [6, 7], and the prevalence of GERD has increased rapidly in Korea [8]. In brief, both GERD and obesity have emerged as important health challenges not only in the West but also in the whole world. Meanwhile, general obesity is measured by body mass index (BMI). Abdominal obesity is measured by waist circumference (WC), and currently, visceral obesity is measured by MRI or CT scan. Abdominal visceral obesity is a more important index of GERD rather than BMI or WC [9–11].

In Korea, voluntary health check-up programs are prevalent, and several packages include upper endoscopy, colonoscopy, abdominal ultrasound, and abdominal CT scan [4, 9, 12]. Under these cultural circumstances, abdominal visceral adipose tissue (VAT) can be measured by a CT scan as well as BMI and WC. Recent studies suggest that VAT is a risk factor for GERD and the area of VAT is correlated with the severity of GERD according to the LA classification of GERD [11, 12].

However, the association between VAT and the sites of esophageal mucosal breaks in subjects with GERD has not been investigated. Esophageal mucosal breaks occur mainly at a site with direct exposure to gastric juice. Thus, the location of mucosal break is likely affected by posture as well as body size or visceral obesity. The aim of this study was to evaluate the influence of a visceral fat area on the site of mucosal breaks in the esophagogastric junction (EGJ).

## 2. Methods and Materials

### 2.1. Study Design and Population

**Figure 1:**
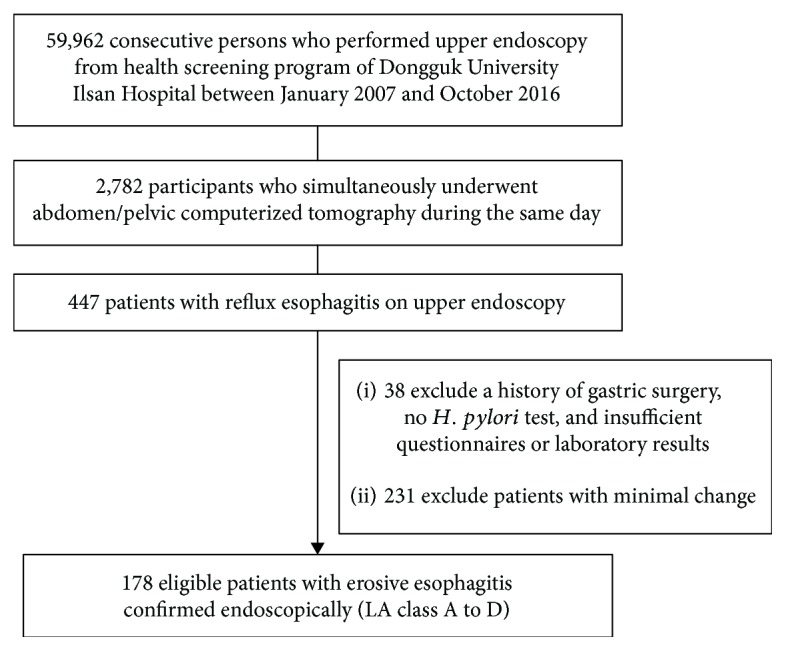
The selection of study population.

The cross-sectional study was conducted in consecutive participants in the voluntary health screening program of Dongguk University Ilsan Hospital in Goyang, Korea, between January 2007 and October 2016. This program comprises overall screening examinations including routine laboratory tests, upper endoscopy, and abdominal sonogram and/or CT, which basically requires overnight fasting. A total of 59,962 subjects underwent screening upper endoscopy during the study period, and 2,782 subjects underwent simultaneous abdomen/pelvic CT during the same day. Among them, 447 patients, diagnosed with reflux esophagitis via upper endoscopy, were eligible for inclusion in the study. Information relating to patients' social history and comorbidity was obtained via established questionnaires based on the screening program. A detailed questionnaire about gastrointestinal symptoms was routinely administered before upper endoscopy. Based on the exclusion criteria such as a history of gastric surgery, lack of *Helicobacter pylori* (*H. pylori*) test results, or insufficient questionnaires or laboratory test results, 38 patients were excluded from the study. We additionally excluded 231 cases with minimal changes such as mild blurring or erythema on the EGJ, and finally, 178 subjects were included (Figure 1). This study was approved by the institutional review board of Dongguk University Ilsan Hospital (2016-136).

### 2.2. Endoscopy

Upper endoscopy was performed using a flexible endoscope (GIF-H260, Olympus Optical Co. Ltd., Tokyo, Japan). Erosive esophagitis was defined by endoscopically confirmed mucosal break on the EGJ. The grading of erosive esophagitis was graded according to the Los Angeles (LA) classification system [13]. The sites of mucosal breaks were described as the posterior wall (PW), lesser curvature (LC), anterior wall (AW), and fundus (FU) sides. In the left lateral decubitus position, the ventral side of the esophagus was always positioned at 12 o'clock of an endoscopy image, which indicates the AW side. Thus, 3 o'clock position (between 2 and 4 o'clock) indicates the LC side, which leads to the LC side of the stomach. The presence of hiatal hernia in the EGJ was determined by a direct view and via J-turn. The hiatal hernia (grades 0-IV) was graded according to Hill's classification [14, 15]. Grade 0 suggested the absence of hiatal hernia whereas grade II or higher, which is consistent with a hiatal width of at least 2 cm, was considered clinically a significant hiatal hernia. The *H. pylori* test using a rapid urease test or histological examination was performed during the endoscopic procedure.

### 2.3. Measurement of Anthropometric Index and Abdominal Obesity

All participants underwent physical measurements including height (cm), weight (kg), and body fat ratio (BFR) (%) using InBody 720 systems (BioSpace, Seoul, Korea). BMI was calculated as weight divided by height in meter squared (kg/m^2^). Abdominal obesity was evaluated by measuring WC, VAT, subcutaneous adipose tissue (SAT), total adipose tissue (TAT), and VAT/SAT ratio based on the method reported previously by our institute [16]; WC (cm) was measured at the midpoint between the lower borders of the rib cage and upper pole of the iliac crest. We used semiautomated image segmentation software implemented in the analysis system 10.0 (Mayo Clinic Foundation, Biomedical Imaging Resource, Rochester, Minnesota, USA). The software threshold was set between -250 and -50 Hounsfield units, which was the specific range for adipose tissue on CT images. VAT was defined as the intra-abdominal fat confined within the rectal sheath. The SPLINE tool was used to demarcate the VAT by drawing a line around the spine and intra-abdominal muscles (rectus abdominis, transverse abdominis, quadratus lumboru, and psoas).

### 2.4. Statistical Analyses

Descriptive statistics for age, anthropometric index, and adipose tissue areas were described as continuous variables (mean ± standard deviation). Other baseline characteristics and endoscopic findings were analyzed as categorical variables. We compared the differences in baseline and clinical findings based on the presence of mucosal breaks in the four directions (PW, LC, AW, and FU) of erosive esophagitis. Independent sample *t*-tests were used to analyze the association between continuous variables and each direction of erosive esophagitis, and chi-square tests for categorical variables. Next, multivariate logistic regression analyses were performed to determine the correlation between anthropometric or abdominal obesity indices with the direction of erosive esophagitis. Each regression model included age, sex, comorbidity, social histories, gastrointestinal symptoms, the presence of hiatal hernia, and *H. pylori* positivity. In addition, we evaluated the risk of esophagitis with LC side mucosal break depending on the quartiles of VAT using logistic regression with adjusted odds ratio (OR) with 95% confidence intervals (CI). All two-sided *P* values < 0.05 were considered significant. Statistical analyses were performed using IBM SPSS Statistics 19.0 (IBM, Armonk, NY, USA).

## 3. Results

### 3.1. Patient Characteristics and Univariate Analyses

Baseline demographics and clinical and endoscopic findings of the 178 patients with erosive esophagitis are described in Table 1. The mean age was 53.5 ± 10.6 yrs (range: 26-88 yrs), and 87.1% were males. The mean levels of the abdominal fat area were 63.2 ± 27.7 cm^2^ for VAT and 80.9 ± 37.8 cm^2^ for SAT. The proportion of patients with two or more gastrointestinal symptoms was 15.2%, and most of the patients belonged to LA classification A or B (98.9%). The LC side was the most common location of esophageal mucosal breaks (58.4%), followed by the PW side (39.3%). The proportion of erosive esophagitis on the AW or FU side was 20.2% (36/178), which was usually accompanied by mucosal breaks on the other sides, and 39.3% of the subjects had grade II or higher grade of hiatal hernia.

In univariate analysis, subjects with erosive esophagitis on the PW side had significantly lower height, weight, and WC than those without mucosal breaks in this direction (Table 2). On the other hand, subjects with LC side break showed significantly higher VAT and TAT as well as higher weight, BMI, and WC than those without LC side break. In terms of gender, a higher proportion of male patients showed the break on the LC side (*P* = 0.014). The presence of hiatal hernia was only significantly associated with the break on the AW side (*P* = 0.038) and showed a marginal significance in relation to the LC side (*P* = 0.057). Other variables such as age, drinking and smoking habits, coffee intake, *H. pylori*, gastrointestinal symptoms, and laboratory findings were not correlated with any location of mucosal breaks.

### 3.2. Abdominal Obesity and the Location of Mucosal Breaks on the EGJ

Anthropometric indices including height, weight, and WC were inversely associated with erosive esophagitis on the PW side (beta coefficient = −0.108, -0.035, and -0.038, respectively) even after adjusting for other baseline and clinical covariates (Table 3). None of the indices correlating with abdominal fat area showed any significant association with PW side mucosal breaks. VAT was still significantly increased in patients with erosive esophagitis on the LC side (beta = 0.014, *P* = 0.034) after adjusting for other covariates (Table 3). The VAT/SAT ratio also varied in patients with LC side mucosal break and others (beta = 1.252, *P* = 0.024). Anthropometric indices, SAT, and TAT showed no significant correlation with LC side mucosal break in multivariate analyses. Male sex did not affect the presence of LC side mucosal break. When the VAT was analyzed as quartiles, the risk of erosive esophagitis on the LC side increased significantly in the VAT of the third and fourth quartiles when compared with that of the first quartile (adjusted odds ratio (OR) 2.90, 95% confidence interval (CI) 1.18 to 7.13, 3rd quartile vs. 1st quartile, *P* = 0.021; OR 3.63, 95% CI 1.44 to 9.10, 4th quartile vs. 1st quartile, *P* = 0.006). Hiatal hernia did not affect the presence of LC side mucosal breaks (adjusted OR = 1.4, *P* = 0.345). Regarding the quartile values of the VAT/SAT, the adjusted OR for the risk of LC side mucosal breaks was 2.91 (95% CI 1.11 to 7.61, 3rd quartile vs. 1st quartile, *P* = 0.030) and 3.02 (95% CI 1.17 to 7.83, 4th quartile vs. 1st quartile, *P* = 0.023) (Table 4).

## 4. Discussion

The present study demonstrated that conventional indices of obesity such as BMI and WC, and visceral obesity were correlated with the presence of erosions on the LC side of EGJ. Higher values of VAT significantly increased the risk of LC erosion. To the best of our knowledge, the association between the sites of mucosal breaks in GERD and visceral obesity has never been studied.

The variation in the site of mucosal breaks according to the VAT area or VAT/SAT ratio may be associated with the body position such as lateral decubitus. Positional changes were probably associated with a considerable redistribution of chime and acid [17, 18]. Body position influenced fasting and postprandial acid reflux. Heartburn is reported frequently in the supine position, and nocturnal reflux is common in complicated GERD [19]. The effect of the lateral position on GERD has been reported but not the site of mucosal breaks per se [20–23]. After infusion of the meal, the LES pressure declined and transient relaxation of the lower esophageal sphincter (TRLES) frequency increased. Acid reflux episodes occurred more than twice as often in the right lateral position [20]. A previous study assessed the effect of posture and meal on reflux composition by impedance monitoring [23]. It showed that the reflux was nearly always liquid-only on the right side whereas reflux associated with the left side and upright position was gas-only or liquid and gas. In the right lateral position, the LC side of the stomach is the most gravity-dependent side [24]. The normal “left-curved” turn of the esophagus into the stomach may be straightened by the effects of gravity while lying in the right lateral decubitus position, and the EGJ may be in a dependent position relative to the gastric pool in that position. The right lateral decubitus position was associated with a greater duration of exposure to pH < 4 and longer esophageal acid clearance compared with the left, supine, and prone. However, the body position did not affect acidity at the gastric cardia and corpus in 10 healthy subjects [21]. TRLES was equally common in both lateral positions in healthy controls [20, 22]. However, another study showed that TRLES occurred more frequently in the right decubitus position in healthy volunteers [21]. These findings are based on different definitions and methods used for the detection of TRLES. The most recent study using manometry, multichannel intraluminal impedance, and scintigraphy demonstrated that TRLES, GER, distension of proximal stomach, and gastric emptying were increased in the right lateral position compared with the left lateral position in subjects with GERD [22].

Interestingly, our study showed that the visible body size measured by height, weight, and WC was rather small in patients with mucosal breaks associated with the PW side compared with others. Therefore, a slightly raised upper body during the supine position may provide symptom relief especially in GERD patients with a normal body size. By contrast, obese patients who generally carry mucosal breaks on the LC are recommended with the left lateral position. Similarly, because the location of mucosal breaks varies depending on the body size and visceral obesity, behavioral instructions related to sleeping posture vary according to the degree of obesity and the site of mucosal breaks.

Some positive correlations were observed between obesity and GERD. First, though the LES pressure in obese subjects was not significantly different compared with those in normal subjects [25], others suggested that the larger BMI has been correlated with the lower LES pressure [26, 27], which is still disputed. However, TRLES is more frequently observed in obese subjects [28]. TRLES is stimulated by gastric distension, and the total exposure of distal esophagus to acid and the proportion of TRLES accompanied by acid reflux were more frequent in obese subjects [29]. TRLES during the 2-hour postprandial period also showed a significantly greater frequency [30]. Second, abdominal obesity increases the intra-abdominal pressure via transmission of the force of adipose tissue to the abdominal cavity, which has been studied using intra-gastric manometry [31–33]. CT scan has been used for adipose tissue measurement to determine the effect of adipose tissue area or volume in GERD. A study using the VAT area cut-off of 100 cm^2^ showed that the level of triglycerides, less than 6 hrs of sleep each night, and the presence of hiatal hernia were associated with GERD in the obese group [11]. Furthermore, the level of the VAT area (per 50 cm^2^) was correlated with the severity of GERD in men, but not in women. However, another study demonstrated that VAT area did not vary between the sexes as a risk factor of GERD [4], whereas the SAT area was not a risk factor for GERD [4, 10]. A recent study measured the ratio of VAT/SAT as well as the VAT area [10]. The VAT/SAT (>0.9) and VAT area (>137.35 cm^2^) were more important than BMI and waist-to-hip ratio as risk factors for GERD. In multivariate analysis, the VAT volume was the only significant factor for GERD, and in both sexes, the VAT volume was associated with GERD [9]. The VAT area and volume were associated with the severity of GERD based on LA classifications A, B, and C [9, 12]. Third, hiatal hernia in obese subjects is significantly associated with esophagitis [3, 34]. The development of hiatal hernia in obese subjects was related to a pressure gradient along the EGJ [35]. However, these studies defined obesity based on WC or BMI. Therefore, there was discrepancy in the definition of obesity based on the adipose tissue area or volume calculated by a CT scan.

The protective effect of *H. pylori* colonization in the stomach against GERD is unknown [36, 37]. In addition, no clinically significant association was observed between *H. pylori* and obesity in a recent study [38]. Our study showed the absence of any correlation between *H. pylori* colonization and the site of mucosal breaks on the EGJ among GERD subjects. However, to determine if *H. pylori* infection affected the GERD location or its severity, various confounding factors affecting *H. pylori* test results need be considered. Further well-designed studies are warranted, including the use of proton pump inhibitors and underlying gastric mucosal conditions such as severe atrophy as well as the history of *H. pylori* eradication.

The strengths of the present study are as follows: First, visceral obesity was measured using a multidetector CT, to calculate the visceral and subcutaneous adipose tissue area as well as BMI and WC. Second, in addition to the LA classification, which was used to define the length of mucosal breaks and circumferential area of EGJ, we suggest the locations of the mucosal breaks as an indicator of GERD severity in obese subjects. Third, we evaluated the presence of *H. pylori* by a CLO test (or Giemsa stain), hiatal hernia during EGD, and individual dietary or lifestyle factors such as smoking and alcohol consumption using questionnaires before health check-up.

The present study has the several limitations. The study population was the participant of a voluntary health screening program which leads to a selection bias. Second, this study was a cross-sectional study. It cannot be observed when the mucosal breaks have developed. Third, the sleeping posture varies from person to person irrespective of obesity. Therefore, it is shortage of evidence that the direction of mucosal breaks is associated with posture. A further study on the relationship between the sleeping posture and the direction of mucosal breaks in obese patients is required. Finally, because the prevalence of erosive esophagitis was significantly higher in Korean men [39], most of the subjects were male in this study setting.

In conclusion, the VAT and the VAT/SAT ratio were significantly correlated with the mucosal breaks in the LC of EGJ. Visceral obesity could influence the location of the mucosal breaks on EGJ.

## Figures and Tables

**(a):** 

Continuous variables	Mean	SD	Range
Age (years)	53.5	10.6	26-88
Height (cm)	170.2	7.6	142-188
Weight (kg)	73.0	14.2	41.1-143.3
Body mass index (BMI) (kg/m^2^)	25.1	4.2	15.1-46.4
Body fat ratio (BFR) (%)	24.8	6.3	11.5-51.1
Waist circumference (WC) (cm)	87.5	11.3	50-134
Visceral adipose tissue (VAT) (cm^3^)	63.2	27.7	6.5-132.8
Subcutaneous adipose tissue (SAT) (cm^3^)	80.9	37.8	14.2-310.5
Total adipose tissue (TAT) (cm^3^)	144.1	56.7	21.8-443.3
VAT/SAT	0.82	0.37	0.18-2.03
HbA1c (mg/dl)	5.8	1.0	4.7-13.2
Total cholesterol (mg/dl)	206.0	44.2	96-496
Triglyceride (mg/dl)	151.1	106.2	24-664
HDL cholesterol (mg/dl)	52.2	15.0	23-127

**(b):** 

Categorical variables	*n*	%
Male sex	155	87.1
Current smoker	55	30.9
Alcohol	85	47.8
Coffee	53	29.8
Diabetes	13	7.3
Hypertension	28	15.7
*H. pylori*-positive	52	29.2
2 symptoms or more	27	15.2
Foreign body sense	13	7.3
Nausea/vomiting	11	6.2
Heartburn	24	13.5
Abdominal discomfort	12	6.7
Epigastric soreness	25	14.0
Dyspepsia	20	11.2
Los Angeles (LA) classification		
A	135	75.8
B	41	23.0
C	2	1.1
D	0	0
Direction of erosion		
Posterior wall side	70	39.3
Lesser curvature side	104	58.4
Anterior wall side	26	14.6
Fundus side	16	9.0
Hiatal hernia grade		
0	86	48.3
I	22	12.4
II	45	25.3
III	24	13.5
IV	1	0.6

HDL: high-density lipoprotein; SD: standard deviation.

**Table 2:** Univariate analyses based on the directions of erosive esophagitis.

Variables	Posterior wall				Lesser curvature				Anterior wall				Fundus			
	Yes (*n* = 70)		No (*n* = 108)		Yes (*n* = 104)		No (*n* = 74)		Yes (*n* = 26)		No (*n* = 152)		Yes (*n* = 16)		No (*n* = 162)	
	Mean	SD	Mean	SD	Mean	SD	Mean	SD	Mean	SD	Mean	SD	Mean	SD	Mean	SD
Age (years)	53.8	10.0	53.3	11.1	53.9	11.6	53.0	9.2	52.5	8.2	53.7	11.0	51.2	7.2	53.8	10.9
Height (cm)	168.0	7.3	171.6	7.5‡	171.0	7.3	168.9	7.9^∗^	171.0	7.3	170.0	7.7	169.3	5.1	170.2	7.8
Weight (kg)	70.1	12.2	74.9	15.1†	75.5	15.4	69.5	11.5‡	73.8	11.5	72.9	14.6	74.3	16.6	72.9	14.0
BMI (kg/m^2^)	24.7	3.8	25.4	4.4	25.8	4.7	24.2	3.1†	25.2	3.3	25.1	4.3	25.8	5.8	25.0	4.0
Body fat ratio (%)	24.7	6.0	24.9	6.5	25.3	6.9	24.2	5.4	24.4	4.6	24.9	6.6	26.1	9.0	24.7	6.0
WC (cm)	85.3	11.2	89.0	11.2†	89.4	12.3	84.9	9.0‡	88.2	7.3	87.4	11.8	86.9	14.2	87.6	11.0
VAT (cm^3^)	58.6	27.0	66.2	27.9^∗^	69.1	26.5	55.0	27.4‡	60.6	23.8	63.6	28.3	59.5	26.1	63.6	27.9
SAT (cm^3^)	78.0	35.0	82.8	39.5	82.9	43.4	78.1	28.1	84.6	24.3	80.3	39.7	90.4	58.4	80.0	35.2
TAT (cm^3^)	136.6	51.6	149.0	59.5	151.9	61.3	133.1	47.7†	145.3	41.0	143.9	59.0	149.9	71.8	143.5	55.2
VAT/SAT	0.79	0.39	0.84	0.35	0.9	0.37	0.72	0.34‡	0.74	0.34	0.84	0.37	0.78	0.48	0.83	0.35

	*N*	%	*N*	%	*N*	%	*N*	%	*N*	%	*N*	%	*N*	%	*N*	%
Male sex	61	87.1	94	87.0	96	92.3	59	79.7†	21	80.8	134	88.2	14	87.5	141	87.0
Current smoker	17	24.3	38	35.2	33	31.7	22	29.7	11	42.3	44	28.9	6	37.5	49	30.2
Alcohol	34	48.6	51	47.2	50	48.1	35	47.3	13	50.0	72	47.4	6	37.5	79	48.8
Coffee	22	31.4	31	28.7	29	27.9	24	32.4	8	30.8	45	29.6	7	43.8	46	28.4
Diabetes	6	8.6	7	6.5	9	8.7	4	5.4	1	3.8	12	7.9	1	6.3	12	7.4
Hypertension	8	11.4	20	18.5	21	20.2	7	9.5^∗^	3	11.5	25	16.4	1	6.3	27	16.7
*H. pylori*-positive	18	25.7	34	31.5	32	30.8	20	27.0	8	30.8	44	28.9	4	25.0	48	29.6
2 symptoms or more	11	15.7	16	14.8	15	14.4	12	16.2	7	26.9	20	13.2^∗^	3	18.8	24	14.8
Hiatal hernia	23	32.9	47	43.5	47	45.2	23	31.1^∗^	15	57.7	55	36.2†	8	50.0	62	38.3
HbA1c ≥ 6.5	13	18.6	14	13.0	16	15.4	11	14.9	3	11.5	24	15.8	1	6.3	26	16.0
TC ≥ 200 (mg/dl)	38	54.3	55	50.9	56	53.8	37	50.0	13	50.0	80	52.6	6	37.5	87	53.7
TG ≥ 150 (mg/dl)	20	28.6	39	36.1	38	36.5	21	28.4	8	30.8	51	33.6	4	25.0	55	34.0
HDL < 40 (M), <50 (F) (mg/dl)	11	15.7	24	22.2	18	17.3	17	23.0	8	30.8	27	17.8	2	12.5	33	20.4

BMI: body mass index; HDL: high-density lipoprotein cholesterol; SAT: subcutaneous adipose tissue; SD: standard deviation; TAT: total adipose tissue; TC: total cholesterol; TG: triglyceride; VAT: visceral adipose tissue WC: waist circumference. *P* value: ^∗^<0.1, ^†^<0.05, ^‡^<0.01.

**Table 3:** Multivariate analyses of anthropometric and abdominal obesity indices for PW and LC erosion.

Variables	PW side			LC side		
	Beta	SE	*P* value	Beta	SE	*P* value
Height (cm)	-0.108	0.031	<0.001	0.030	0.027	0.257
Weight (kg)	-0.035	0.016	0.032	0.027	0.015	0.081
Body mass index (kg/m^2^)	-0.037	0.045	0.414	0.072	0.047	0.128
Body fat ratio (%)	0.003	0.029	0.911	0.033	0.030	0.273
Waist circumference (cm)	-0.038	0.018	0.033	0.027	0.017	0.112
Visceral adipose tissue (VAT) (cm^3^)	-0.011	0.007	0.104	0.014	0.007	0.034
Subcutaneous adipose tissue (SAT) (cm^3^)	-0.003	0.005	0.539	0.002	0.005	0.662
Total adipose tissue (TAT) (cm^3^)	-0.004	0.003	0.235	0.004	0.003	0.194
VAT/SAT	-0.332	0.517	0.533	1.252	0.553	0.024

The other covariates (age, sex, diabetes, hypertension, smoking, alcohol, coffee, symptoms, hiatal hernia, and *H. pylori*) are adjusted for these regressions. PW: posterior wall; AW: anterior wall; SE: standard error.

**Table 4:** Adjusted odds ratio for LC side mucosal breaks.

Variables	OR	95% CI	*P* value
VAT (cm^3^)			
1st quartile (<44.6)	1		
2nd quartile (44.7-64.3)	2.28	0.94-5.49	0.067
3rd quartile (64.4-82.1)	2.90	1.18-7.13	0.021
4th quartile (>82.2)	3.63	1.44-9.10	0.006

VAT/SAT ratio			
1st quartile (<0.54)	1		
2nd quartile (0.55-0.76)	1.45	0.59-3.54	0.418
3rd quartile (0.77-1.02)	2.91	1.11-7.61	0.030
4th quartile (>1.03)	3.02	1.17-7.83	0.023

The other covariates (age, sex, diabetes, hypertension, smoking, alcohol, coffee, symptoms, hiatal hernia, and *H. pylori*) are adjusted for these regressions. LC: lesser curvature; OR: odds ratio; CI: confidence interval; VAT: visceral adipose tissue; SAT: subcutaneous adipose tissue.

## Data Availability

The data used to support the findings of this study are available from the corresponding author upon request.
